# Editorial: Plant secondary metabolite biosynthesis

**DOI:** 10.3389/fpls.2024.1477551

**Published:** 2024-08-21

**Authors:** Xiang Pu, Naoki Kitaoka, Carlos E. Rodríguez-López, Shilin Chen

**Affiliations:** ^1^ College of Science, Sichuan Agricultural University, Ya’an, China; ^2^ Research Faculty of Agriculture, Hokkaido University, Sapporo, Japan; ^3^ Tecnologico de Monterrey, Escuela de Ingeniería y Ciencias, Monterrey, Mexico; ^4^ Tecnologico de Monterrey, The Institute for Obesity Research, Monterrey, Mexico; ^5^ Institute of Herbgenomics, Chengdu University of Traditional Chinese Medicine, Chengdu, China

**Keywords:** plant, secondary metabolite, biosynthetic pathway, gene characterization, synthetic biotechnology

## Introduction

Plant secondary metabolites (PSM) are a diverse group of compounds that contribute to many important biological and ecological functions. They are synthesized by plants to interact with the biotic and abiotic environments, playing roles in plant defense, growth, and development (Erb and Kliebenstein, 2020). Additionally, PSM have widespread applications in human industries, including food additives, cosmetics, dyes, insecticides, and drugs. The biosynthesis of PSM is complex and dynamic, with more than one million PSM estimated to be present in terrestrial and aquatic plants (Afendi et al., 2012). Despite their diversity, plants produce limited quantities of PSM in a metabolic cost-saving way. This has limited their commercial production, and the overexploitation of source plants has raised concerns about their sustainability and highlighted the need for advanced research.

Recent advances in genomics (Siadjeu and Pucker, 2023), transcriptomics (Voelckel et al., 2017), metabolomics (Li et al., 2024), and other omics technologies have revolutionized our understanding of plant biology, enabling the discovery of new PSM and their biosynthetic pathways. Functional genomics approaches, such as genome-wide association studies (Luo et al., 2020), transcriptome analysis (Liu et al., 2021), and gene editing (Nasti and Voytas, 2021), have facilitated the identification and characterization of genes involved in the biosynthesis of PSM. Metabolic engineering and synthetic biology approaches have enabled the manipulation of plant secondary metabolism to improve the yield and quality of specific metabolites of interest or to produce them in heterologous cultures (Zhang et al., 2022). These advances have created new opportunities for the sustainable production and utilization of PSM.

This Research Topic on Plant Secondary Metabolite Biosynthesis illustrates a comprehensive and up-to-date view of the biosynthesis, regulation, and biotechnological production of PSM, and to promote interdisciplinary and cross-disciplinary research collaborations in this field for sustainable and efficient utilization of these valuable compounds.

## Identifying and characterizing novel genes involved in the biosynthesis of PSM

Combining bioinformatics method, gene quantitative analysis, and evolutionary analysis, Wang et al. retrieve 103 *BAHD* genes from the Ginseng Genome Data resource. Phylogenetic analysis indicates that these *BAHD* genes are clustered in three clades. Most *PgBAHD*s contain cis-acting elements associated with abiotic stress response and plant hormone response. Among them, 34 *PgBAHD*s are clustered with genes that display malonyl transferase activity. Seven *PgBAHD*s (*PgBAHD4*, *PgBAHD45*, *PgBAHD65*, *PgBAHD74*, *PgBAHD90*, *PgBAHD97*, and *PgBAHD99*) are designated as the potential candidates involved in the biosynthesis of malonyl ginsenosides based on co-expression analysis. To fully elucidate the biosynthetic pathways of orobanchol derivatives in Fabaceae plants, Homma et al. probe the metabolomic pathways downstream of orobanchol in cowpea, barrel medic, and pea via substrate feeding experiment after different enzyme inhibitor (fluridone, uniconazole-P, and prohexadione) treatment. Subsequently, *DOX* and *BAHD* acyltransferases responsible for converting orobanchol to their derivatives are mined from the public dataset and screened using co-expression analysis. Enzymatic assays of heterologously expressed proteins reveal that the DOX in barrel medic converts orobanchol to medicaol, the DOX and BAHD acyltransferase in pea convert orobanchol to fabacol and acetylated fabacol, the favacol acetyltransferase and its homolog in cowpea acetylate orobanchol. These findings shed light on the molecular mechanisms underlying the structural diversity of strigolactones.

Hendrickson et al. characterize series of terpene synthases (TPS) from *Medicago truncatula*, a model legume. Thirty-nine *MtTPS* candidates are obtained from the Mt4.0v1 genome in Phytozome (tps://phytozome-next.jgi.doe.gov). They assess the *MtTPS* activity using a modular metabolic engineering system in *E. coli* and characterize the resultant metabolite using GC-MS. Analyses of the resultant metabolite reveal the production of an assortment of sesquiterpenes. This work also establishes the gene-to-metabolite relationships for sesquiterpene synthase in *M. truncatula*. To explore the glycosylation step for rutin biosynthesis in *Solanum melongena*, Gan et al. identify 195 UDP-glycosyltransferase (UGT) candidate genes from the *S. melongena* genome V4.0 (ttps://solgenomics.net). These *UGT* genes are classified into 17 subgroups and the members of Groups A, B, D, E, and L are associated with flavonol biosynthesis. A hierarchical clustering analysis of gene expression profiles reveals that the expression patterns of *SmUGT* genes in Clusters 7-10 are closely related to those of rutin biosynthetic pathway genes. Among them, *SmUGT89B2* is verified to encode the final enzyme in rutin biosynthesis via virus-induced gene silencing and transient overexpression assay in eggplant. Rosmarinic acid (RA) is one of the major bioactive components of *Prunella vulgaris*. Yan et al. identified 51 RA biosynthesis-related genes from the chromosome-level genome of *P. vulgaris*. Bioinformatic analyses and gene expression pattern indicate that 17 of them could be involved in RA biosynthesis. *In vitro* enzymatic assay reveals that PvRAS3 catalyzes the condensation of *p*-coumaroyl-CoA and caffeoyl-CoA with pHPL and DHPL, and PvRAS4 only catalyzes the condensation of *p*-coumaroyl-CoA with pHPL and DHPL. Generation of *pvras3* homozygous mutants through CRISPR/Cas9 technology and subsequent chemical compound analysis confirm that PvRAS3 is the main enzyme catalyzing the condensation of acyl donors and acyl acceptors during RA biosynthesis in *P. vulgaris*. This work supports the existence of at least four RA biosynthetic pathways, with the role of *PvRAS4* appears minor in this plant.

## Developing and employing innovative biotechnological techniques to improve the yield of PSM

Zheng et al. assemble a high-quality genome of *Fagopyrum dibotrys*. Based on evolutionary relationship analysis, the authors speculate that *F. dibotrys* has originated in the high-altitude Tibetan Plateau region. The homologues of genes involved in the biosynthesis of flavonoids are annotated. This study could reveal the genetic background and facilitate the cultivation of high-yielding *F. dibotrys* varieties. Li et al. employ single-cell RNA sequencing to profile the transcriptomes of protoplasts derived from *Gynostemma pentaphyllum* shoot apexes and leaves. Examining gene expression patterns across various cell types reveal that genes related to gypenoside biosynthesis are predominantly expressed in mesophyll cells. They also explore the impact of transposable elements (TE) on *G. pentaphyllum* transcriptomic landscapes. This study not only provides new insights into the spatiotemporal organization of gypenoside biosynthesis and TE activity in shoot apexes and leaves, but also offers valuable cellular and genetic resources for improving the yield of gypenoside in *G. pentaphyllum*.

*Mentha spicata* (spearmint) possesses peltate glandular trichomes (PGT) where valuable essential oils are produced. Reddy et al. identify a novel non-canonical Aux/IAA gene, *MsIAA32*, from spearmint, which lacks the TIR1-binding domain and regulates the development of PGT. Using yeast two-hybrid studies, two canonical Aux/IAAs (*MsIAA3*, *MsIAA4*) and an ARF (*MsARF3*) are identified as the preferred binding partners of *MsIAA32*. The possible role of *MsIAA32* in non-glandular trichome formation is confirmed by ectopic expression in Arabidopsis. Undoubtedly, identifying new gene targets controlling PGT numbers in spearmint will provide new ways to increase volatile/scented PSM production. Dendrobine, a noteworthy alkaloid found in *Dendrobium nobile*, possesses valuable pharmaceutical potential. Sarsaiya et al. develop innovative approaches to enhance dendrobine production by utilizing endophytic fungi. Using test bottles (EGTB), the experimental group (12-month-old *D. nobile* seedling), co-cultured with *Trichoderma longibrachiatum* (MD33), displays a 2.6-fold denrobine increase (1804.23 ng/ml) compared to the control group (685.95 ng/ml). Co-culturing *D. nobile* seedlings with *T. longibrachiatum* MD33 in the temporary immersion bioreactor systems (EGTIBS) leads to a substantial 9.7-fold dendrobine increase (4415.77 ng/ml) compared to the control (454.01 ng/ml) after 7 days. Scaling up the TIBS approach for commercial dendrobine production could provide an accessible platform for dendrobine production.

*Phoebe zhennan* is widely cultivated in China and the price of nanmu wood is expensive. Yang et al. explore the composition and content of essential oil and fragrance compounds in *P. zhennan* wood at different tree ages. The yield of essential oil from 30a wood is significantly greater than that from 10a and 80a wood. In total, 596 (LC) and 76 (GC) features are annotated using chromatography-coupled mass spectrometry in the essential oil and wood. This research determines that the main components of the wood fragrance are sesquiterpenoids. The types and relative contents of sesquiterpenoids from wood increase with tree age. These results suggest that choosing wood from trees of a suitable age will significantly improve the yield of essential oil. Qin et al. successfully improve the seed tocopherol concentration by altering chlorophyll metabolism. They find that RNAi suppression of *CHLSYN* coupled with homogentisate phytyltransferase (HPT) overexpression increase tocopherol concentration by more than two-fold in Arabidopsis seeds. Additional three-fold increase in seed tocopherol are observed by engineering homogentisate production via overexpression of bacterial *TyrA*, which encodes chorismate mutase/prephenate dehydrogenase. They further increase total tocochromanol concentration by overexpression of the gene for hydroxyphenylpyruvate dioxygenase. These biofortification approaches shift metabolism towards increased amounts of tocotrienols relative to tocopherols. This study provides a theoretical basis for genetic improvement of total tocopherol concentrations in green oilseeds.

## Plant secondary metabolite biosynthesis, the road ahead

The number of PSM is still expanding due to the diversity of plant species and the rapid advances in analytical technology. Undoubtedly, PSM has served as a natural compound resource for human industries. But there is a contradiction between the insatiable human needs for PSM and the existing chemical-based production methods, such as plant source-dependent extraction and sophisticated chemical synthesis. Metabolic engineering and synthetic biology approaches have been verified as one of the best ways for PSM manufacture. These innovative biotechnological techniques depend on fully elucidating the biosynthetic pathway and biochemically charactering the gene elements responsible for PSM biosynthesis one by one. In recent years, characterization and heterologous reconstitution of the biosynthetic pathway for several valuable PSM including artemisinic acid, thebaine, colchicine, and baccatin III have been reported. These findings are intriguing and encouraging for researchers focusing on PSM biosynthesis. However, there is still a long way to go. A huge number of biosynthetic steps for PSM remain unknown. Thousands of gene elements responsible for PSM biosynthesis remain uncharacterized. Modern omics technologies, novel gene manipulation approaches, and artificial intelligence technology has expanded our knowledge on PSM biosynthesis and will facilitate the biomanufacture of valuable PSM.

## References

[B1] Afendi F. M. Okada T. Yamazaki M. Hirai-Morita A. Nakamura Y. Nakamura K.. (2012). KNApSAcK family databases: integrated metabolite–plant species databases for multifaceted plant research. Plant Cell Physiol. 53, e1. doi: 10.1093/pcp/pcr165 22123792

[B2] Erb M. Kliebenstein D. J. (2020). Plant secondary metabolites as defenses, regulators, and primary metabolites: the blurred functional trichotomy. Plant Physiol. 184, 39–52. doi: 10.1104/pp.20.00433 32636341 PMC7479915

[B3] Li Y. H. Hirai M. Y. Nakamura Y. (2024). Plant metabolomics. J. Exp. Bot. 75, 1651–1653. doi: 10.1093/jxb/erae047 38481104

[B4] Liu M. Y. Yang L. Cai M. M. Feng C. Zhao Z. M. Yang D. P.. (2021). Transcriptome analysis reveals important candidate gene families related to oligosaccharides biosynthesis in *Morinda officinalis* . Plant Physiol. Biochem. 167, 1061–1071. doi: 10.1016/j.plaphy.2021.09.028 34601436

[B5] Luo T. T. Xia W. Gong S. F. Mason A. S. Li Z. Y. Liu R.. (2020). Identifying vitamin E biosynthesis genes in *Elaeis guineensis* by genome-wide association study. J. Agric. Food Chem. 68, 678–685. doi: 10.1021/acs.jafc.9b03832 31858793

[B6] Nasti R. A. Voytas D. F. (2021). Attaining the promise of plant gene editing at scale. Proc. Natl. Acad. Sci. U.S.A. 118, e2004846117. doi: 10.1073/pnas.2004846117 34050019 PMC8179185

[B7] Siadjeu C. Pucker B. (2023). Medicinal plant genomics. BMC Genomics 24, 429. doi: 10.1186/s12864-023-09542-8 37528364 PMC10391748

[B8] Voelckel C. Gruenheit N. Lockhart P. (2017). Evolutionary transcriptomics and proteomics: Insight into plant adaptation. Trends Plant Sci. 22, 462–471. doi: 10.1016/j.tplants.2017.03.001 28365131

[B9] Zhang J. Hansen L. G. Gudich O. Viehrig K. Lassen L. M. M. Schrübbers L.. (2022). A microbial supply chain for production of the anti-cancer drug vinblastine. Nature 609, 341–347. doi: 10.1038/s41586-022-05157-3 36045295 PMC9452304

